# The dynamics of microbial community and flavor metabolites during the acetic acid fermentation of *Hongqu* aromatic vinegar

**DOI:** 10.1016/j.crfs.2022.10.002

**Published:** 2022-10-04

**Authors:** Wen-Long Li, Shan-Gong Tong, Zi-Yi Yang, Yan-Qin Xiao, Xu-Cong Lv, Qi Weng, Kui Yu, Gui-Rong Liu, Xiao-Qing Luo, Tao Wei, Jin-Zhi Han, Lian-Zhong Ai, Li Ni

**Affiliations:** a Food Nutrition and Health Research Center, School of Advanced Manufacturing, Fuzhou University, Jinjiang, Fujian, 362200, PR China; b Institute of Food Science and Technology, College of Biological Science and Engineering, Fuzhou University, Fuzhou, Fujian, 350108, PR China; c Fujian Salt Industry Group Co., Ltd., Fuzhou, Fujian, 350001, PR China; d Fujian Minyan Food Technology Co., Ltd., Sanming, Fujian, 365500, PR China; e School of Medical Instruments and Food Engineering, University of Shanghai for Science and Technology, Shanghai, 200093, PR China

**Keywords:** *Hongqu* aromatic vinegar, T, raditional fermentation, Microbial dynamics, F, lavor metabolites, C, orrelation analysis

## Abstract

In this study, we investigated the dynamics of microbial community and flavor metabolites during the traditional fermentation of *Hongqu* aromatic vinegar (HAV) and subsequently explored the potential relationship between microbiota and flavor metabolites. The microbiome analysis based on high-throughput sequencing (HTS) of amplicons demonstrated that *Lactobacillus*, *Acetobacter* and *Clostridium* were the dominant bacterial genera, while *Alternaria*, *Candida*, *Aspergillus* and *Issatchenkia* were the dominant fungal genera during the acetic acid fermentation (AAF) of HAV. A total of 101 volatile flavor compounds were identified through gas chromatography-mass spectrometry (GC-MS) during HAV fermentation, including esters (35), alcohols (17), aldehydes (11), acids (11), ketones (7), phenols (10), and others (10). Redundancy analysis (RDA) was used to reveal the correlation between microbiota and volatile flavor compounds. *Lactobacillus* and *Acetobacter* were the two bacterial genera that have the great influence on the production of volatile flavor components in HAV. Among them, *Lactobacillus* was positively correlated with a variety of ethyl esters, while *Acetobacter* positively contributed to the formation of several organic acids. Furthermore, the non-volatile metabolites were detected by ultra-high-performance liquid chromatography with quadrupole time-of-flight mass spectrometry (UPLC-QTOF/MS). A total of 41 dipeptides were identified during HAV fermentation, and most of them may have sensory characteristics and biological activities. RDA showed that *Aspergillus*, *Epicoccum*, *Issatchenkia*, *Candida* and *Malassezia* were the most influential fungal genera on non-volatile metabolites. In particular, *Epicoccum* was first reported in *Hongqu* vinegar and showed a positive correlation with the production of various organic acids. In conclusion, this study provides a scientific basis for understanding the flavor generation mechanism of HAV, and may be valuable for developing effective techniques to select suitable strains to improve the flavor quality of HAV.

## Highlights

• Microbial community in *Hongqu* aromatic vinegar was investigated by high-throughput sequencing.
• The key flavor metabolites during the acetic acid fermentation of HAV were selected through PLS-DA.
• *Lactobacillus*, *Acetobacter* and *Clostridium* were the predominant bacterial genera in HAV fermentation.
• *Alternaria*, *Candida*, *Aspergillus* and *Issatchenkia* were the predominant fungal genera in HAV fermentation.
• Redundancy analysis (RDA) revealed the correlation between microbiota and flavor metabolites.

## Graphical abstract

**Figure:**
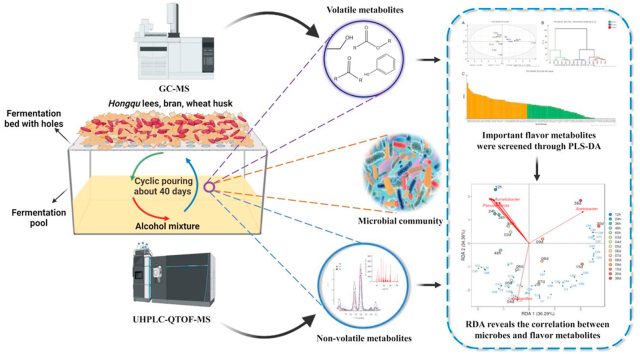

## 1 Introduction

Chinese cereal vinegar has a production history of more than 3000 years, and its brewing technology has become an important part of Chinese traditional food culture (Guan et al., 2014; Tesfaye et al., 2002). As one of the four predominant vinegars in China, Fujian *Hongqu* vinegar (also named *Monascus* vinegar) is well known for its unique flavor quality (Jiang et al., 2019). In addition, because *Hongqu* (Monascus) is used as a starter culture, *Hongqu* vinegar may have potential health benefits such as lipid-lowering (Song et al., 2020), cholesterol-lowering (Heber et al., 1999) and antioxidant effect (Tseng et al., 2006). A recent study showed that oral administration of *Hongqu* vinegar can ameliorate hyperlipidemia by regulating the relevant protein expressions and modulating the gut microbiota composition (Song et al., 2020).

*Hongqu* aromatic vinegar (HAV) is a kind of *Hongqu* vinegar brewed from *Hongqu* lees, bran, wheat husk and *Hongqu* rice wine with mother vinegar as fermentation starter by special solid-liquid layered fermentation process. The fermentation tanks are separated by hollow plate, the upper layer is filled with a mixture of *Hongqu* lees and rice husk, while the lower layer is filled with *Hongqu* rice wine (approximate 6% vol alcohol)*.* When the solid raw materials in the upper layer begin to heat up spontaneously due to the action of microorganisms, the stock solution in the lower layer is pumped to the upper layer for recycling and reflux, and the whole reflux process lasts about 40 days at a frequency of 3 times a day*.* During the traditional fermentation of HAV, the spontaneous aggregation of microbiota is the key factor for flavor shaping. In addition, the environmental factors can also drive microbes to produce a large amount of organic acids and other flavor compounds through various metabolic pathways during the acetic acid fermentation (Shi et al., 2022). Therefore, the microbial flora and its metabolism largely determine the aroma and taste characteristics of HAV.

Previously, many studies have been conducted on the microbial community in traditional Chinese vinegar and its potential relationship with flavor metabolites, and the core functional microorganisms in the traditional fermentation process have been revealed. For example, in the acetic acid fermentation (AAF) stage of Zhenjiang aromatic vinegar, *Aspergillus* and *Alternaria* were revealed as the dominant fungal genera, while *Lactobacillus* and *Acetobacter* dominated the bacterial community and were considered as the core microbiota with important contributions to the formation of various flavor components (Wang et al., 2016). Similarly, *Acetobacter* and *Lactobacillus* were revealed as the key dominant bacterial genera in Shanxi aged vinegar, which are closely related to the production of various flavor components (Zhu et al., 2018). Nevertheless, the correlation between microbial flora and flavor metabolites during the brewing process of HAV has not been elucidated yet. Due to lack of the theoretical indication, maintaining quality with a stable and consistent flavor between different batches of HAV products has proved to be considerably difficult when the traditional fermentation processes are carried out in an open and non-sterile environment. Therefore, it is necessary to investigate the dynamic variations of microbes and metabolites during the entire brewing process to further clarify the relationship between microbes and flavors.

Thus, this study investigated the potential correlations between the dominant microbiota and key flavor metabolites during the acetic acid fermentation of HAV. Furthermore, it is meaningful to explore the key functional microbes responsible for the flavor formation of *Hongqu* vinegar. This study will provide the theoretical basis for steering and improving the quality of *Hongqu* vinegar in industrial manufacturing scale.

## 2 Materials and methods

### 2.1 Sample collection

All the samples of HAV were collected from Fuquanchun Vinegar Factory in Yongchun County, Quanzhou city, Fujian province of China. In order to ensure the uniformity of the vinegar brewing mass, samples were collected after reflux mixing of the lower layer fermentation broth. The sampling time was set as 0.5, 1, 1.5, 2, 2.5, 3, 4, 5, 6, 7, 8, 9, 15, 30 and 38 days of brewing. After being collected, the samples were immediately centrifuged at 8000 rpm under 4 °C for 10 min. The supernatant and precipitate were separated and loaded into 50 mL and 2 mL sterile centrifuge tubes, respectively, and stored at −80 °C for subsequent experiments.

### 2.2 Determination of physicochemical parameters

The pH is directly measured by a pH meter (FiveEasy Plus™, Mettler Toledo, China). The determination methods of reducing sugar, alcohol, and total acid according to the previously described methods (Huang et al., 2022; Zhang et al., 2020). Additionally, the organic acids were analyzed using a UHPLC (UltiMate 3000, Thermo Fisher Scientific, USA) at 215 nm with a Syncronis aQ-C18 (4.6 × 250 mm, 5 μm) column. The operating conditions were as follows: the mobile phase was prepared by mixing KH_2_PO_4_ solution (0.02 M) with acetonitrile at a ratio of 98:2, eluted at a flow rate of 1.0 mL/min for 35 min. The concentrations of organic acids in the fermentation samples were calculated by linear regression analysis based on the peak area.

### 2.3 Detection of volatile flavor compounds

The profile of volatile flavor compounds was determined using HS-SPME combined with GC-MS (7890-B/5977A, Agilent Technology, USA). The sample treatment and the instrument operation were performed according to (Zhao et al., 2022) with some modifications. Briefly, the raw vinegar sample was collected and diluted 10 times with purified water as the test sample, then 10 μL of 2-octanol (10 mg/L) was added into the sample as internal standard. Thereafter, the GC-MS equipped with HP-INNOWAX capillary column (30.0 m × 0.25 mm × 0.25 mm, Agilent Technology, USA) was used to measure the volatile flavor compounds of each sample.

### 2.4 The extraction of non-volatile metabolites

Initially, 100 μL sample was transferred to a EP tube of 2 mL, and 400 μL extract solution (acetonitrile: methanol = 1:1) containing an isotopically-labelled internal standard mixture was added. After 30 s vortex, the samples were sonicated for 10 min in an ice-water bath. Then the samples were incubated at −40 °C for 1 h and centrifuged at 12000 rpm (RCF = 13800×*g*) for 15 min at 4 °C. The supernatant was taken for 400 μL and transferred to an unused tube and dried in a vacuum concentrator at 37 °C. Then, the dried samples were re-dissolved with 100 μL of 50% acetonitrile by sonication for 10 min in ice-water bath. The constitution was then centrifuged at 13000 rpm (RCF = 16200×*g*) for 15 min at 4 °C, and 80 μL supernatant was transferred to a glass vial for LC/MS analysis. The control sample was prepared by mixing the equal aliquots mentioned above except for the supernatants from all the samples.

### 2.5 Non-volatile metabolites detection and data analysis

The non-volatile metabolites of each sample were determined using UHPLC (1290 Infinity LC, Agilent Technologies, USA) coupled to a quadrupole time of-flight (TripleTOF 6600, AB Sciex, USA) mass spectrometer. The UHPLC was equipped with a UPLC BEH Amide column (2.1 × 100 mm, 1.7 μm, Waters) and the procedure of UHPLC was followed the method described previously (Chen et al., 2022), except the QTOF-MS analysis with slight modification. ESI source conditions were set as follows: gas 1 60 psi, gas 2 60 psi, curtain gas 35 psi, and the source temperature was set as 600 °C, declustering potential as 60 V. Meanwhile, the Ion Spray Voltage Floating (ISVF) was set as 5000 V or −4000 V in positive or negative modes, respectively. The data analysis was conducted following that: the MS raw data (.wiff) files were converted to the mzXML format by ProteoWizard, and processed by R package XCMS, which consists of peak deconvolution, alignment and integration. While, the minfrac and cut off were set as 0.5 and 0.3 respectively. Moreover, in-house MS2 database was applied for metabolites identification.

### 2.6 DNA sequencing and bioinformatic analysis

Total DNA was extracted from the precipitate of *Cupei* according to the protocol of rapid DNA extraction kit (MN NucleoSpin 96 Soi, Germany). The extracted DNA was used as a template, and the V3–V4 region of bacterial 16S rDNA and ITS1 region of fungal ITS-5.8S rDNA were amplified by 338F (5′-ACT CCT ACG GGA GGC AGC A-3)/806-R (5′-GGA CTA CHV GGG TWT CTA AT-3′) and ITS5-1737-F (5′-GGA AGT AAA AGT CGT AAC AAG G-3′)/ITS2-2043-R (5′-GCT GCG TTC TTC ATC GAT GC-3′), respectively. Then, the sequencing library was constructed and sequenced according to our previous studies (Huang et al., 2019). After the sequencing data were spliced and filtered according to the overlapping relationship, the quality of the sequences was controlled and screened. Raw sequencing reads were quality-filtered and analyzed using FLASH software (v1.2.7) and QIIME software (v1.8.0). Operational taxonomic unit (OTU) clustering analysis was performed at sequence similarity of 97% based on the UPARSE algorithm using USEARCH software (v10.0). The bacterial OTU sequences were annotated using the SILVA/16S rDNA database (Quast et al., 2013) by a QIIME-based wrapper of the RDP-classifier (v.2.2) (Cole et al., 2009). The OTU sequences of fungal ITS were clustered using the UNITE database by USEARCH (ver 10.0) and aligned by the BLAST algorithm.

### 2.7 Statistical analysis

The website of OmicStudio (https://www.omicstudio.cn/tool) and the heat map package in the R software were used to visualize the distribution of volatile components by heat map. Additionally, SIMCA-14.1 software was used for principal component analysis (PCA) to evaluate the clustering trend of different components. Partial least squares discriminant analysis (PLS-DA) modeling was also carried out using SIMCA-14.1 software to reveal the key differential components quickly and accurately in various samples.

## 3 Results and discussion

### 3.1 Physicochemical parameters of vinegar brewing

**Fig. 1:**
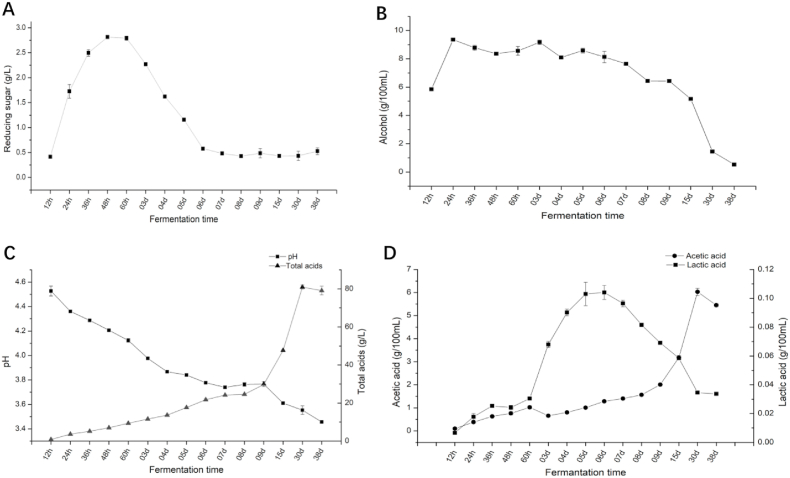
Changes of physiochemical parameters during HAV fermentation. Reducing sugar (A), Alcohol (B), Total acids and pH (C), Acetic acid and lactic acid (D).

The physicochemical parameters are the universal indicators used to monitor the AAF process of HAV (Fig. 1). The content of reducing sugar was first increased from 0.125 g/L to 2.792 g/L (12 h–60 h), then was decreased to 0.577 g/L (6 d), and almost remained constant during the subsequent fermentation (Fig. 1A). Moreover, the alcohol content reached 9.353 g/100 mL (12 h–24 h), after that, the alcohol level changed following a stepwise downward trend, till approaching 0 g/100 mL on the 38th day (Fig. 1B). While, the total acid was moderately increased from 0.263 g/L to 30.025 g/L in the first 9 days, then raised sharply and reached the highest concentration on the 30th day, and then consequently declined slightly to 79.108 g/L on the 38th day (Fig. 1C). Meanwhile, the variation trend of pH was opposite to that of total acid, and reached the lowest point of 3.46 on the 38th day (Fig. 1C). Acetic acid and lactic acid are the main organic acids in vinegar, and their content and proportion greatly affect the taste of vinegar (Wu et al., 2017; Zhu et al., 2018). In present study, the variation trend of acetic acid was as similar as total acid, reaching the top point of 5.452 g/100 mL on the 30th day, and then decreased slightly (Fig. 1D). Simultaneously, the content of lactic acid approached the peak of 0.104 g/100 mL on the 6th day, and then declined to 0.034 g/100 mL on the 38th day (Fig. 1D). Lactic acid can be converted to acetic acid, which may account for the decrease in lactic acid in this study. (Chai et al., 2020).

### 3.2 The evolution of the microbial community

**Fig. 2:**
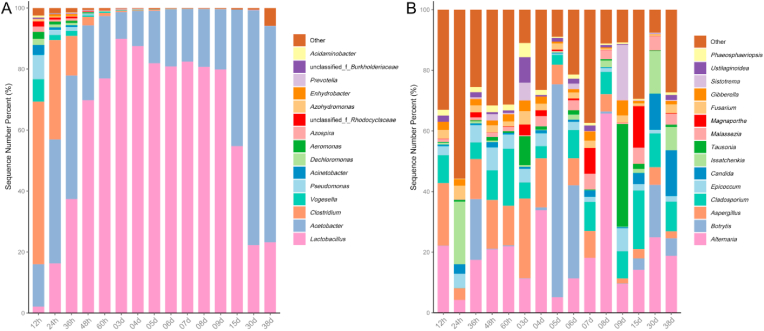
Relative abundance of the microbial genera during HAV fermentation process. Bacteria (A), Fungi (B).

HAV as a well-known traditional brewed-food, microorganisms play a decisive role in the fermentation. The microbial community succession during acetic fermentation of HAV was measured through amplicon high-throughput sequencing. As shown in Fig. 2A, *Clostridium*, *Acetobacter*, *Lactobacillus*, *Vogesella*, *Acinetobacter*, *Pseudomonas*, *etc*. were determined as the main bacteria genera in the AAF stage of HAV. With the progress of fermentation, *Lactobacillus* and *Acetobacter* became the absolute dominant bacteria, and the sum of their relative abundance exceeded 90% after 48 h of fermentation, which is similar to the study of Shanxi aged vinegar (Zhu et al., 2018). This phenomenon may be because environmental stress factors such as ethanol and acetic acid inhibit the growth of many microorganisms, thus affecting microbial diversity (Zheng et al., 2018). The abundance of *Lactobacillus* began to decrease from the 9th day, while the abundance of *Acetobacter* showed a synchronous increase. This phenomenon may be due to the accumulation of acetic acid gradually inhibited the growth of *Lactobacillus*. Interestingly, *Lactobacillus* still maintained a certain abundance at the fermentation endpoints, which may be attributed to some species of *Lactobacillus* with strong acid tolerance, such as *Lactobacillus acetotolerans*, which was found to increase in the late fermentation stage of Zhenjiang aromatic vinegar (Chai et al., 2020). Moreover, *Clostridium* was abundant in the initial stage of fermentation, which may be derived from fermentation materials. According to a previous study, *Clostridium* is considered to have the potential to degrade cellulose and hexose, which can provide substrates for the metabolism of other microorganisms (Wu et al., 2017). As for fungal community, no obvious succession rule was found during acetic acid fermentation of HAV (Fig. 2B). *Alternaria*, *Candida, Aspergillus* and *Issatchenkia* were the dominant genera and were present throughout the fermentation stage. Among them, it was reported that *Candida* is the dominant bacterial genus in other fermented cereal foods (such as sourdough bread), which contributes to the formation of carbon dioxide, lactic acid, acetic acid and ethanol (Brandt et al., 2004). In addition, *Alternaria* and *Aspergillus* are the common fungi in fermented foods, which have also been reported in previous studies on vinegars (Wang et al., 2015, 2021).

### 3.3 Changes of volatile flavor compounds during HAV brewing

**Fig. 3:**
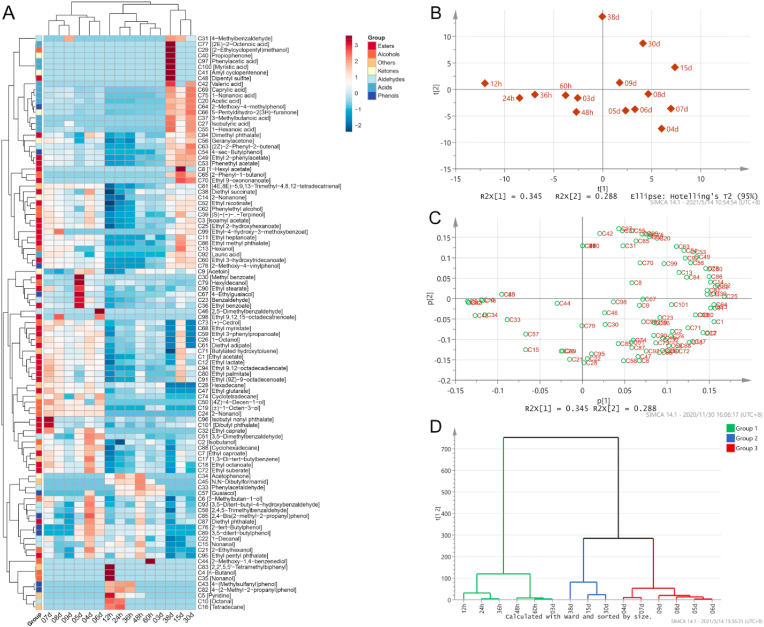
Changes of volatile flavor components during HAV fermentation process. Heatmap of the abundance of volatile flavor components (A). Score plot (B), loading plot (C) and hierarchical cluster plot (D) of the abundance of volatile flavor components based on PCA.

The profile of volatile flavor compounds is a key index to accurately reflect the quality and normal fermentation of HAV. In this study, a total of 101 volatile flavor compounds in HAV were identified using SPME-GC-MS, including 35 esters, 17 alcohols, 11 acids, 11 aldehydes, 7 ketones, 10 phenols, and 10 others (Fig. 3A). As shown in the PCA score plot (Fig. 3B), the fermentation samples presented a counter-clockwise arrangement as the fermentation time progressed. Fermentation samples at different time points were obviously separated, indicating that the compositions of volatile components were significantly different. Meanwhile, the PCA loading plot was also shown in Fig. 3C, which helps us understand the characteristics of the main volatile compounds in the samples at different fermentation stages (Fig. 3C). Moreover, the results of hierarchical cluster based on the similarity of volatile flavor substances also clearly showed that the acetic acid fermentation samples of HAV could be clustered into three groups, namely group I (12h-03d), group II (15d-30d), and group III (04d-09d), indicating that the volatile characteristics changed with fermentation time (Fig. 3D).

During HAV fermentation, 17 alcohols were detected (Fig. 3A). At the beginning of fermentation, n-butanol [C4] and nonanol [C35] were abundant, which may be attributed to the fermentation substrate. In addition, the major alcohols consisting of 3-methylbutan-1-ol [C6], (±)-1-Octen-3-ol [C19], 2-ethylhexanol [C21], 2-nonanol [C24], 1-Octanol [C26], 2-methoxy-1,4-benzenediol[C44], (4Z)-4-decen-1-ol [C50], phenylethyl alcohol [C62], (+)-cedrol [C73], hexyldecanol [C79] increased firstly and then decreased during AAF. Alcohols are important substrates of acetic acid fermentation, which are usually produced by the conversion of sugars by hexokinase, glucokinase and aldose 1-epimerase produced by yeast, or as a result of decarboxylation and subsequent reduction of ketoacids (Al-Dalali et al., 2020). Furthermore, alcohols are suggested to be contributors to the aromatic profiles of vinegar due to their unique odors. For example, several alcohols found in this study were also considered as the characteristic aromatic alcohols in Zhenjiang aromatic vinegar, such as phenylethyl alcohol (floral), 2-ethylhexanol (floral, honey) and 3-methylbutan-1-ol (fruity, caramel) (Yu et al., 2012).

Esters are also regarded as the important volatile flavor compounds in HAV, which can provide vinegar with pleasant odor (Fig. 3A). As the fermentation going on, a variety of esters were increased gradually, involving ethyl acetate [C1], isoamyl acetate [C3], ethyl caproate [C7], ethyl lactate [C12], ethyl benzoate [36], phenethyl acetate [C53], methyl benzoate [C30], dipentyl sulfite [C48], dimethyl phthalate [C84], ethyl 2-phenylacetate [C49], ethyl nicotinate [C52], ethyl 3-hydroxytridecanoate [C60], ethyl 9-oxononanoate [C70], ethyl palmitate [C80], ethyl 9,12-octadecadienoate [C94]. Generally, esters are derived from the reaction between acids and alcohols, and most of esters have floral and fruit aroma, which can impart HAV with desirable flavor (Dumitriu et al., 2017; Marín et al., 2002). For example, phenylethyl acetate, which is widely used as rose flavoring agent in food industry, and is one of the main esters in HAV (Carvalho et al., 2017). However, the concentration of some esters decreased slightly after the 15th day, especially ethyl esters including ethyl acetate [C1], ethyl caprate [C32], ethyl glutarate [C47], ethyl myristate [C68], diethyl adipate [C61], ethyl suberate [C72]. The decreases in the concentrations of ethyl esters may be due to their volatility or the shift in esterification equilibrium that occurs at lower concentrations of alcohols (Guerrero et al., 2008).

Meanwhile, almost all the volatile organic acids were steadily increased during AAF. Among them, acetic acid [C20] is the main organic acid, which is the main source of sharp acid taste of HAV. Besides, 1-hexanoic acid [C55], caprylic acid [C69], 1-nonanoic acid [C75], lauric acid [C92], isobutyric acid [C27], valeric acid [C42], myristic acid [C100], phenylacetic acid [C97], 3-methylbutanoic acid [C37] and (2E)-2-octenoic acid [C77] were also detected in HAV (Fig. 3A). Multiple organic acids can reconcile the sharp taste of acetic acid and improve the sensory quality of HAV.

Other volatile flavor compounds involving phenols, aldehydes, and ketones were also detected in HAV (Fig. 3A). The great majority of aldehydes and ketones exhibit nut aroma and fruit aroma, and may be derived from the degradation of amino acids and unsaturated fatty acids, and Maillard reaction (Zhao et al., 2020). 2-Nonanone [C14], 4-methylbenzaldehyde [C31], (2Z)-2-phenyl-2-butenal [C63] and acetoin [C9] were found to present an increasing trend in AAF of HAV. Among them, acetoin (3-hydroxy-2-butanone), with a pleasant yogurt odor and a fatty creamy butter taste and is often used as food additive to optimize the flavor of products (Xiao and Lu, 2014). Moreover, it was also discovered that the content of guaiacol [C57] decreased in the later stage of AAF, while other volatile phenols including 4-sec-butylphenol [C54], 2-methoxy-4-methylphenol [C64], 2-methoxy-4-vinylphenol [C78], 4-ethylguaiacol [C67] increased obviously. These phenols can enhance the richness of aroma even at low level, and they are considered to be one of the principal aroma substances in wine, *Baijiu*, soy sauce and vinegar (Gallardo-Chacon and Karbowiak, 2015; Sun et al., 2022; Wang et al., 2020).

### 3.4 Correlation analysis of microbiota and volatile flavor components

**Fig. 4:**
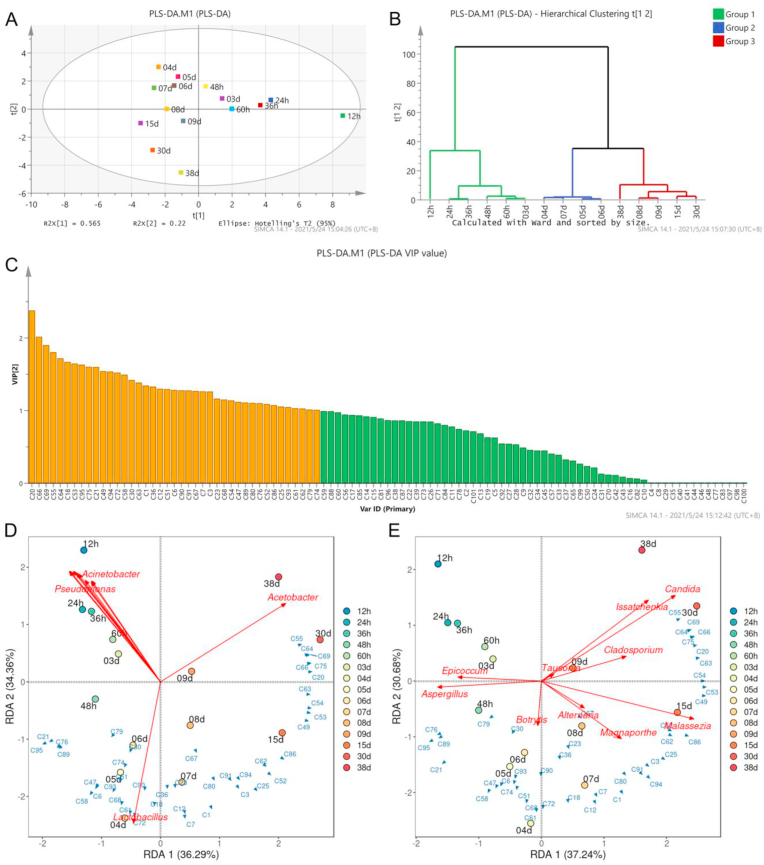
Partial least squares discriminant analysis (PLS–DA) of volatile flavor components during HAV fermentation (A). Hierarchical cluster plot (B) and VIP score plot (C) of volatile components based on PLS-DA. RDA between dominant microbial community (bacteria (D) and fungi (E)) and key non-volatile metabolites.

PLS-DA model was established to screen the key volatile components with significant variation to further explore the potential correlation between volatile flavor components and microbiota in the brewing process. The score plot of PLS-DA clearly showed that the volatile components of HAV changed gradually with the progress of brewing (Fig. 4A). According to the hierarchical clustering of the composition of volatile flavor components, the fermentation samples can be clustered into three categories (Fig. 4/B). In order to determine the key volatile flavor components that changed significantly during the fermentation process, the volatile flavor components with *VIP* (variable importance for the projection) score >1 were selected as key components to further explore the potential association with microorganism (Fig. 4C).

In this study, RDA was carried out to further reveal the correlation between microbial communities (the top 10 dominant bacteria/fungi) and key volatile flavor components during HAV fermentation. The RDA results showed that *Lactobacillus* and *Acetobacter* were the critical bacterial genera that affected the formation of key volatile flavor components in HAV fermentation (Fig. 4D). Noteworthy, *Lactobacillus* is positively related to a variety of aromatic substances, including ethyl suberate [C72], ethyl octanoate [C18], diethyl adipate [C61], ethyl myristate [C68], 3,5-ditert-butyl-4-hydroxybenzaldehyde [C93], ethyl caproate [C7], ethyl acetate [C1], ethyl lactate [C12], ethyl stearate [C90], ethyl benzoate [C36], ethyl palmitate [C80] and benzaldehyde [C23] *etc*. Interestingly, most of the above substances belong to ethyl esters, which can enrich the aroma composition of HAV. Correspondingly, a previous study also reported that *Lactobacillus* is positively correlated with a variety of ethyl esters (including ethyl acetate, ethyl phenylacetate, *etc*.) in light-flavor *Baijiu* brewing (Huang et al., 2020). As for *Acetobacter*, the dominant bacterial genus at the end of HAV brewing, was positively correlated with 2-methoxy-4-methylphenol [C64], 1-hexanoic acid [C55], caprylic acid [C69], 5-pentyldihydro-2(3H)-furanone [C66], 1-nonanoic acid [C75] and acetic acid [C20], suggesting that *Acetobacter* may contribute to the formation of various organic acids, similar results have been found in a previous study on Zhejiang rosy vinegar (Fang et al., 2021).

It is well known that fungi play a crucial role in fermented food due to the abundant and diverse enzymes (Copetti, 2019). However, there are few studies on the function of fungal community during vinegar fermentation, which may be related to the low fungal biomass during AAF (Huang et al., 2022; Wang et al., 2016). However, under environmental interference, some minority of microbial taxa can greatly affect the interaction between local dominant microorganisms, thus affecting their metabolic characteristics (Xue et al., 2018). Therefore, this study also explored the potential relationship between dominant fungi and volatile flavor components in HAV (Fig. 4E). According to RDA, *Issatchenkia* and *Candida* were positively correlated with 1-hexanoic acid [C55], caprylic acid [C69], 2-methoxy-4-methylphenol [C64], 5-pentyldihydro-2(3H)-furanone [C66], 1-nonanoic acid [C75], acetic acid [C20] and (2Z)-2-phenyl-2-butenal [C63] and 4-sec-butylphenol [C54]. As typical non*-Saccharomyces* yeasts, *Issatchenkia* and *Candida* can produce a variety of characteristic flavor substances and enhance the flavor quality of fermented food. Previous study showed that *Issatchenkia* and *Candida* could produce β-glucosidase to increase the contents of terpenes, esters and fatty acids and thus enhance the flavor complexity and character of wines (Zhang et al., 2020; Thongekkaew et al., 2018). In addition, co-fermentation or sequential fermentation based on *Issatchenkia* and *Saccharomyces cerevisiae* enriches the aroma of wine (Shi et al., 2019). Surprisingly, two phenols (2-tert-butylphenol [C76] and 3,5-di-tert-butylphenol [C89]) showed positive correlations with *Aspergillus* in this study. Previously, *Aspergillus* has also been identified as a key fungal genus in other traditional Chinese fermented foods (Jiao et al., 2022).

### 3.5 Changes of non-volatile metabolites during HAV brewing

**Fig. 5:**
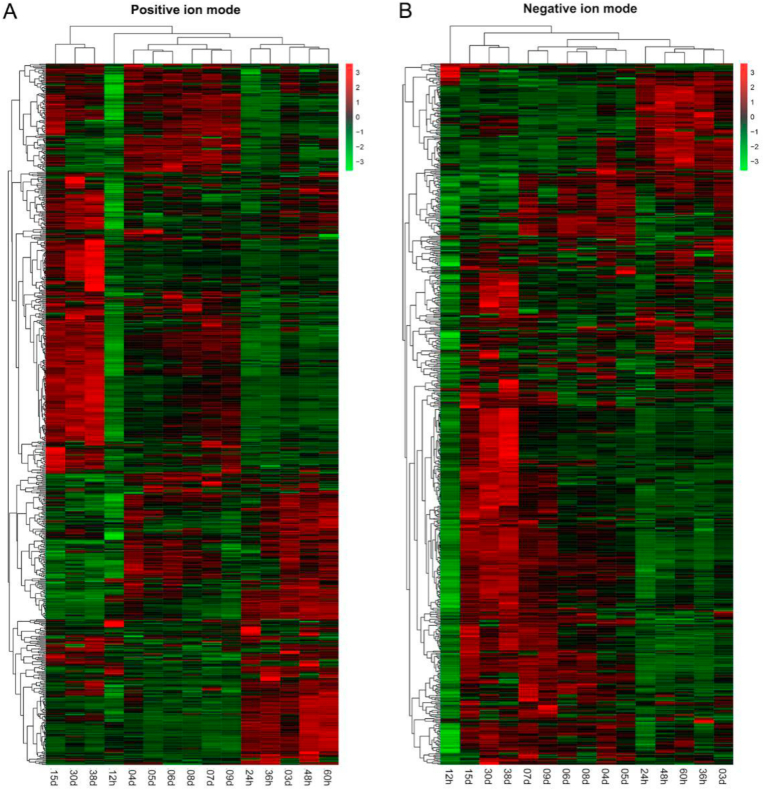
Heat maps of the abundance of non-volatile components detected by UPLC-QTOF/MS under the positive ion mode (A) and the negative ion mode (B).

**Fig. 6:**
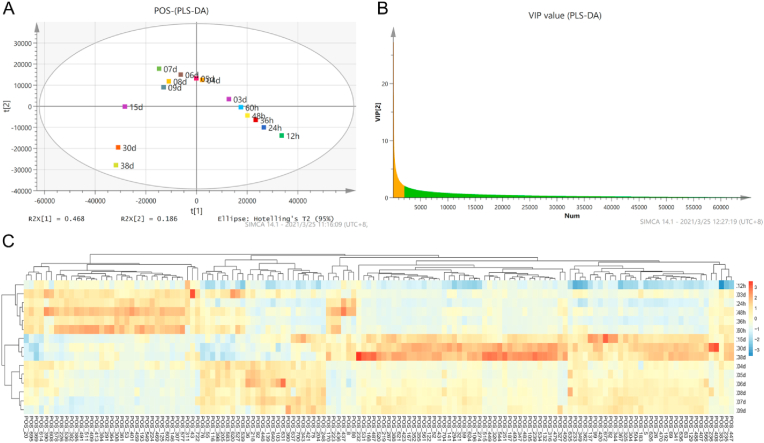
PLS–DA of the abundance of non-volatile flavor components during HAV fermentation in positive ion mode. Score plot based on PLS-DA (A), VIP plot based on PLS-DA (B). Heatmap of the abundance of non-volatile flavor components with VIP >2 (C).

**Fig. 7:**
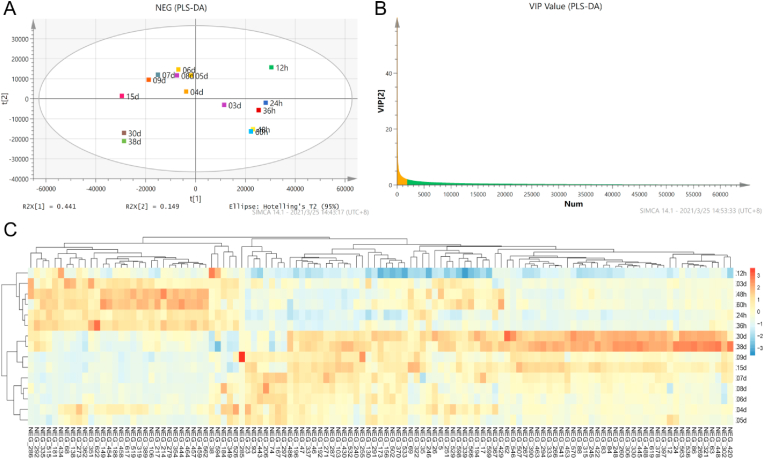
PLS–DA of the abundance of non-volatile flavor compounds during HAV fermentation in negative ion mode. Score plot based on PLS-DA (A), VIP plot based on PLS-DA (B). Heatmap of the abundance of non-volatile flavor components with VIP >2 (C).

The non-volatile metabolites during HAV brewing were detected by UHPLC-QTOF/MS. A total of 732 and 639 non-volatile metabolites were identified in positive and negative ion modes, respectively (Fig. 5). PCA can reflect the overall variability of non-volatile metabolites during AAF. Both in positive and negative ion modes, the result of PCA showed that the compositions of non-volatile metabolites changed obviously during the brewing of HAV. According to the hierarchical clustering of the composition of non-volatile components, the fermentation samples can be clustered into three categories (Supplementary material-1: Fig. S1 & Fig. S2). The PLS-DA was used to further screen out key non-volatile metabolites that changed significantly during HAV brewing. PLS-DA score plots clearly showed that the composition of non-volatile components detected in the positive and negative ion modes changed gradually with the progress of HAV brewing, especially after 9th days. (Fig. 6A & Fig. 7A). To reveal the key non-volatile metabolites that changed significantly during HAV fermentation, the metabolites with *VIP* socre >2 were screened as key metabolites to further explore their potential association with microbial community (Figs. 6B and 7B).

A total of 141 non-volatile metabolites with VIP score more than 2 were obtained in the positive ion mode and a heat map was drawn based on the trend of their signal values (Supplementary material-2 & Fig. 6C). During the brewing process of HAV, the content of small molecular carbohydrates changed significantly, D-fructose [POS390] and D-mannose [POS401] both increased first and then decreased. The hydrolysis of starch in fermentation raw materials may be the main reason for the increases of D-fructose and D-mannose content, which can then enter the tricarboxylic acid cycle through glycolysis to produce organic acids and other flavor compounds (Chen et al., 2022), and can also provide energy and carbon skeleton for microbial growth and material metabolism (Jiang et al., 2020). In addition, nine amino acids were found, including L-histidine [POS291], L-tyrosine [POS509], ornithine [POS36], D-proline [POS69], L-phenylalanine [POS5], L-isoleucine [POS626], DL-serine [POS488], L-valine [POS437], L-citrulline [POS407]. Different amino acids have different taste characteristics, which can enrich the taste of HAV. For example, L-histidine, L-phenylalanine and L-tyrosine were described as bitterness, while the L-valine and DL-serine were considered sweetness (Xue et al., 2022). These abundant free amino acids in the vinegar are helpful to improve the sensory characteristics of HAV. Additionally, a total of 41 dipeptides were identified during AAF, most of which had different sensory properties and bioactive function (Supplementary material-1: Table S1). There are 24 dipeptides associated to savour, including 16 bitter peptides, 4 umami peptides, 2 sour peptides and 2 salty enhancing peptides. Also, dipeptides involved in biological activities were also identified, activities were also identified, including 29 containing DPP IV inhibitor, 23 containing ACE inhibitor, 17 with both functions mentioned above, and 6 with antioxidant function (Supplementary material-1: Table S1). The dipeptidyl peptidase IV (DPP IV) inhibitors were regarded as potential agents for the treatment of type 2 diabetes (Hikida et al., 2013). In this study, Ile-Pro (*IC*_50_ = 0.41 mM), Phe-Pro (*IC*_50_ = 0.36 mM), Phe-Phe (*IC*_50_ = 0.73 mM) and Val-Arg (*IC*_50_ = 0.82 mM) were reported to possess strong DPP IV inhibitor function (Adriana et al., 2017; Hatanaka et al., 2012; Nongonierma and Fitzgerald, 2013). Besides, some dipeptides have the activity of inhibiting angiotensin converting enzyme (ACE) related to anti-hypertension, and exhibit the potential of anti-hypertension (Wei et al., 2021). These results indicated that a variety of dipeptides are produced during HAV fermentation, which can enrich the bioactive function of HAV.

In the negative ion mode, 118 substances with VIP scores >2 were screened (Supplementary material-2 & Fig. 7B), and the heat map was drawn according to their signal value (Fig. 6C). Several sugars and acids were found to increase and then decrease during HAV brewing. For instance, alpha-D-glucose [NEG319], L-malic acid [NEG458], cis-aconitate [NEG279] and D-erythrulose [NEG68] were observed to increase during the first 3 days of fermentation and then decrease. These substances may be derived from the starch hydrolysis in raw materials, and then can be consumed through glycolysis, pyruvate metabolism and tricarboxylic acid cycle to provide precursors and energy for the synthesis of lipids, amino acids and fatty acids. In addition, deoxyuridine [NEG106], guanosine [NEG292] and L-tyrosine [NEG389] and glycyl-L-leucine [NEG454] were also abundant during the first 5 days, which may be derived from protein and nucleic acid degradation. As expected, the contents of various organic acids increased significantly after 15 days of fermentation. 2-hydroxy-2,4-pentadienoate [NEG86], benzoic acid [NEG488], alpha-ketoglutarate [NEG448], L-pyroglutamic acid [NEG333], azelaic acid [NEG445], (3R)-3-isopropenyl-6-oxoheptanoate [NEG306], 3-hydorxy-3-methylglutaric acid [NEG422], sarcosine [NEG390], D-glucono-1,5-lactone [NEG453], 4-hydroxyhexan-3-one [NEG62] and L-proline [NEG546] gradually increased after 15 days and reached the highest signal value at the end of fermentation, which may directly contribute to the flavor of HAV. Also, several bioactive substances including tulipalin B [NEG536] and vanillin [NEG397] were detected. Tulipalin B, known as β-hydroxy-α-methyl-γ-butyrolactone, is a bioactive substance that can be used as an antibacterial agent (Jing and Chen, 2015). And vanillin has been shown to reduce hippocampal neuronal death caused by global cerebral ischemia (Kim et al., 2007), as well as can pass through the blood-brain barrier to perform significant neuroprotective effect by reducing oxidative stress damage in *vivo* and in *vitro* (Lan et al., 2019). However, some health-threaten substances were also detected during HAV brewing. For example, phenylethylamine [POS561] and tyramine [POS341] were captured, which are biogenic amines usually formed based on the catalysis of the corresponding amino acids by amino acid decarboxylase. Low intakes of biogenic amines can promote growth and metabolism and eliminate free radicals, while excessive intakes may lead to various health problems involving headache, hypotension and palpitation, which are harmful to the nervous and cardiovascular system (Zhang et al., 2019). According to previous studies, it is considered that the metabolic pathway of by-products of some organisms including *Lactobacillus* and *Pseudomonas*, especially *Lactobacillus*, is the main reason for biogenic amines (Barbieri et al., 2019). In the future, we may devote ourselves to research on how to control the production of these harmful substances to improve the health performance of fermented food.

### 3.6 Correlation analysis of microbial community and non-volatile metabolites

**Fig. 8:**
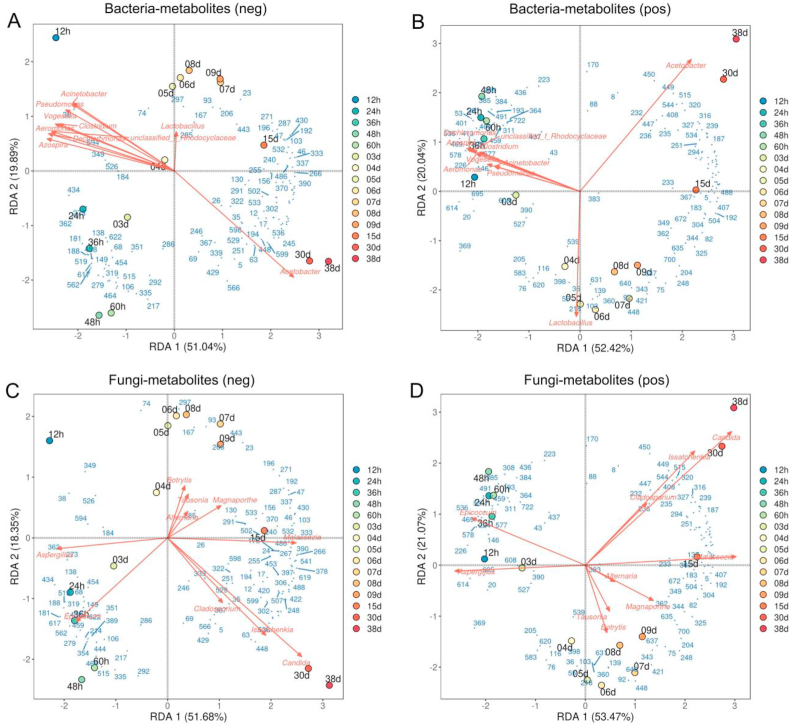
RDA between dominant bacteria and key non-volatile metabolites in negative ion mode (A) and positive ion mode (B). RDA between dominant fungi and the key non-volatile metabolites in negative ion mode (C) and positive ion mode (D).

In this study, RDA was carried out to further reveal the correlation between the dominant dominant bacteria/fungi and the key non-volatile metabolites during HAV fermentation (Fig. 8). The RDA of microbial community and non-volatile metabolites showed that *Lactobacillus* and *Acetobacter*, the two dominant bacterial genera of HAV fermentation, are positively correlated with a variety of non-volatile metabolites, and exhibit correlation characteristics different from other bacteria genera (Fig. 8A and B). Meanwhile, non-volatile metabolites positively correlated with *Lactobacillus* and *Acetobacter* also indirectly indicated their contribution to the flavor and nutrition of HAV. For example, *Lactobacillus* has been revealed to be positively associated with several functional substances, such as atropine [POS139], nicotinamide [POS218], harmaline [POS76] and pantetheine [NEG206] (Fig. 8A and B). Among them, atropine has been reported to be used in clinical treatment of myopia (Yam et al., 2021). Nicotinamide, a precursor of nicotinamide adenine dinucleotide (NAD+), has a high safety profile even at considerable doses and can attenuate obesity, aging, hearing loss, and vision loss (Brown et al., 2014; Mendelsohn and Larrick, 2014; Poljsak and Milisav, 2018; Trammell et al., 2016). Harmaline has inhibitory effect on bovine milk xanthine oxidase, and shows great potential in the treatment of gout (Linani et al., 2021). Pantetheine is a precursor of Coenzyme A biosynthesis, which is an important substance for microbial metabolism. In addition, many amino acids and their derivatives, including 3,4-dihydroxy-L-phenylalanine [NEG285], 3-methyl-L-tyrosine [NEG297], calligonine [NEG167], phenylacetic acid [NEG93], quinaldic acid [POS55], L-formylkynurenine [POS103], ornithine [POS36], tauroursodeoxycholic acid [POS631], N-acetyl-L-leucine [POS398], folinic acid [POS116], Pro-Arg [POS620], D-glucono-1,5-lactone [POS583] and Phe-Val [POS205], also show positive correlations with the abundance of *Lactobacillus* (Fig. 8A and B). Similar to *Lactobacillus*, *Acetobacter* was positively correlated with a variety of organic acids, amino acids and dipeptides, mainly including Tyr-Pro [POS236], Pro-Thr [POS408], 4-aminobutyric acid [POS515], Leu-Tyr [POS447], Pro-Thr [POS408], His-Thr [POS187], alpha-ketoglutarate [NEG448], N5-(L-1-carboxyethyl)-L-ornithine [NEG63], (3R)-beta-Leucine 5, 2-hydroxyphenylacetic acid [NEG194], trans-2-hydroxycinnamic acid [NEG529], 2-oxoadipic acid [NEG367] (Fig. 8A and B). A previous study had shown that acetic acid stress can up-regulate *glnA* (encoding glutamine synthetase) of *Acetobacter*, and increase the intracellular concentration of ammonium to resist acid damage to the internal environment (Xie et al., 2021). Meanwhile, the up-regulation of *glnA* promotes the synthesis of ammonia, glutamate and glutamine, which are nitrogen donors for the synthesis of amino acids (Xie et al., 2021). Amino acids can be further condensed into dipeptides, which may be the reason for the positive correlation between *Acetobacter* and many amino acids and dipeptides in this study. Other bacterial genera, including *Clostridium*, *Acinetobacter* and *Vogesella*, show positive correlations with oleic acid [NEG38], 5-hydroxymethyluracil [NEG526], N-acetyl-beta-alanine [NEG184], D-mannose [POS401], Val-Arg [POS409], L-arabinono-1,4-lactone [POS311], 5-L-glutamyl-L-alanine [POS361], and these metabolites were abundant at the initial stage of HAV fermentation (Fig. 8A and B).

According to the results of RDA, *Aspergillus*, *Epicoccum*, *Issatchenkia*, *Candida* and *Malassezia* were considered to have great influence on metabolites (Fig. 8C and D). In short, L-galactono-1,4-lactone [NEG362], pyruvaldehyde [NEG273], 18-hydroxyoleate [NEG138], orotate [NEG434], Ala-His [POS614], cytidine [POS20], Thr-Phe [POS527], Leu-Gln [POS608], 5,6-dihydrothymine [POS695] were analyzed to be positively associated with *Aspergillus*. A previous study suggested that *Aspergillus* can promote the utilization of starch in vinegar, then increase the accumulation of amino acids and other substances to facilitate the production of vinegar (Liu et al., 2020). Interestingly, several organic acids such as L-malic acid [NEG458], cis-aconitate [NEG279], citrate [NEG351], mevalonic acid [NEG188], glyoxylate [NEG464] showed a positive association with *Epicoccum*. The fungi from the genus *Epicoccum* are mainly known for their use as biocontrol agents against phytopathogens and for their ability to produce many secondary metabolites with potential biotechnological applications, such as antioxidant, anticancer and antimicrobial compounds (Braga et al., 2018). To the best of our knowledge, *Epicoccum* is the first reported presence in a *Hongqu* vinegar brewing system in this study. Its existence also indirectly proved that there were abundant fungal resources in *Hongqu* vinegar brewing environment. *Candida* and *Issatchenkia* are non*-Saccharomyces* yeast and can contribute positively to the quality of fermented foods (Ciani et al., 2010). In this study, *Candida* and *Issatchenkia* show positive influence on the formation of tulipalin B [NEG536], alpha-ketoglutarate [NEG448], (3R)-beta-leucine [NEG5], coumaryl acetate [NEG429], trans-2-hydroxycinnamic acid [NEG529], 4-aminobutyric acid [POS515], Pro-Thr [POS408], caffeine [POS544], nicotinate [POS449], 3-dimethylallyl-4-hydroxybenzoate [POS316], albuterol [POS232] and Tyr-Pro [POS236], *etc*. *Malassezia* was generally recognized as a pathogenic fungus causing skin infection in previous studies (Hamdino et al., 2022). In the present study, *Malassezia* was related to a variety of amino acids including L-pyroglutamic acid [NEG333], sarcosine [NEG390], L-threonine [NEG266], L-phenylalanine [POS5] and L-citrulline [POS407], *etc*. Thses results suggest that the biological functions of microorganisms may be different depending on the living environment.

## 4 Conclusion

As one typical representative of Chinese traditional vinegar, HAV is characterized for its aroma, taste, nutrient and bioactive function. The residues of fermentation substrate and the microbial metabolites accumulated in the vinegar provide the material basis for the unique flavour of HAV. In this study, the profile of volatile compounds, variation of microbial community and non-volatile flavour ingredients were determined and clarified, during the acetic acid fermentation of HAV. Furthermore, the correlations between the microbial community and the profile of volatile compounds, as well as the non-volatile components, were analyzed and elucidated, respectively. The results of this study can be helpful to control and steer the quality of HAV manufacture, and provide the theoretical basis for the investigation of other similar fermentation foods.

## CRediT authorship contribution statement

**Wen-Long Li:** Methodology, Investigation, Writing – original draft. **Shan-Gong Tong:** Data curation, Software, Visualization. **Zi-Yi Yang:** Investigation, Conceptualization. **Yan-Qin Xiao:** Investigation, Writing – original draft. **Xu-Cong Lv:** Writing – review & editing, Supervision, Project administration. **Qi Weng:** Resources. **Kui Yu:** Design and factory management. **Gui-Rong Liu:** Factory management. **Xiao-Qing Luo:** Factory management. **Tao Wei:** Factory sampling. **Jin-Zhi Han:** Software, Writing – review & editing, Supervision, Conceptualization. **Lian-Zhong Ai:** Supervision, Conceptualization. **Li Ni:** Supervision, Validation, Project administration.

## Declaration of competing interest

The authors declare that they have no known competing financial interests or personal relationships that could have appeared to influence the work reported in this paper.

## Data Availability

Data will be made available on request.

## References

[bib1] Adriana C.N., Pádraigín A., HarnedyMartina A., O'Keeffe R.J., Fitz Gerald (2017). Bioactive peptides from Atlantic salmon (*Salmo salar*) with angiotensin converting enzyme and dipeptidyl peptidase IV inhibitory, and antioxidant activities. Food Chem..

[bib2] Al-Dalali S., Zheng F., Sun B., Zhou C., Li M., Chen F. (2020). Effects of different brewing processes on the volatile flavor profiles of Chinese vinegar determined by HS-SPME-AEDA with GC-MS and GC-O. LWT--Food Sci. Technol..

[bib3] Barbieri F., Montanari C., Gardini F., Tabanelli G. (2019). Biogenic amine production by lactic acid bacteria: a Review. Foods.

[bib4] Braga R.M., Padilla G., Araújo W.L. (2018). The biotechnological potential of *Epicoccum* spp.: diversity of secondary metabolites. Crit. Rev. Microbiol..

[bib5] Brandt M., Hammes W., Gänzle M.G. (2004). Effects of process parameters on growth and metabolism of *Lactobacillus sanfranciscensis* and *Candida humili*s during rye sourdough fermentation. Eur. Food Res. Technol..

[bib6] Brown K., Maqsood S., Huang J., Pan Y., Harkcom W., Li W., Sauve A., Verdin E., Jaffrey S. (2014). Activation of SIRT3 by the NAD(+) precursor nicotinamide riboside protects from noise-induced hearing loss. Cell Metabol..

[bib7] Carvalho B., Holt S., Ben S., Brandão R.L., Foulquié M., Maria R., Thevelein J. (2017). Identification of novel alleles conferring superior production of rose flavor phenylethyl acetate using polygenic analysis in Yeast. mBio.

[bib8] Chai L., Shen M., Sun J., Deng Y., Lu Z., Zhang X., Shi J., Xu Z. (2020). Deciphering the d-/l-lactate-producing microbiota and manipulating their accumulation during solid-state fermentation of cereal vinegar. Food Microbiol..

[bib9] Chen L., Li D., Rong Y. (2022). Fermentation mechanism of ginkgo rice wine using an ultra-high-performance liquid chromatography–quadrupole/time-of-flight mass spectrometry based metabolomics method. J. Food Compos. Anal..

[bib10] Ciani M., Comitini F., Mannazzu I., Domizio P. (2010). Controlled mixed culture fermentation: a new perspective on the use of non*-Saccharomyces* yeasts in winemaking. FEMS Yeast Res..

[bib11] Cole J.R., Wang Q., Cardenas E., Fish J., Chai B., Farris R.J., Kulam-Syed-Mohideen A., McGarrell D., Marsh T., Garrity G., Tiedje J. (2009). The ribosomal database project: improved alignments and new tools for rRNA analysis. Nucleic Acids Res..

[bib12] Copetti M. (2019). Yeasts and molds in fermented food production: an ancient bioprocess. Curr. Opin. Food Sci..

[bib13] Dumitriu G., López de Lerma N., Zamfir C., Cotea V., Peinado R. (2017). Volatile and phenolic composition of red wines subjected to aging in oak cask of different toast degree during two periods of time. LWT--Food Sci. Technol..

[bib14] Fang G.Y., Chai L.J., Zhong X.Z., Jiang Y.J. (2021). Deciphering the succession patterns of bacterial community and their correlations with environmental factors and flavor compounds during the fermentation of Zhejiang rosy vinegar. Int. J. Food Microbiol..

[bib15] Gallardo-Chacon J., Karbowiak T. (2015). Sorption of 4-ethylphenol and 4-ethylguaiacol by suberin from cork. Food Chem..

[bib16] Guan B., Zhao J., Cai M., Lin H., Yao L., Sun L. (2014). Analysis of volatile organic compounds from Chinese vinegar substrate during solid-state fermentation using a colorimetric sensor array. Anal. Methods.

[bib17] Guerrero E.D., Chinnici F., Natali N., Marín R.N., Riponi C. (2008). Solid-phase extraction method for determination of volatile compounds in traditional balsamic vinegar. J. Separ. Sci..

[bib18] Hamdino M., Saudy A.A., El-Shahed L.H., Taha M. (2022). Identification of *Malassezia* species isolated from some *Malassezia* associated skin diseases. J. Med. Mycol..

[bib19] Hatanaka T., Inoue Y., Arima J., Kumagai Y., Usuki H., Kawakami K., Kimura M., Mukaihara T. (2012). Production of dipeptidyl peptidase IV inhibitory peptides from defatted rice bran. Food Chem..

[bib20] Heber D., Yip I., Ashley J.M., Elashoff D.A., Elashoff R.M., Go V. (1999). Cholesterol-lowering effects of a proprietary Chinese red-yeast-rice dietary supplement. Am. J. Clin. Nutr..

[bib21] Hikida A., Ito K., Motoyama T., Kato R., Kawarasaki Y. (2013). Systematic analysis of a dipeptide library for inhibitor development using human dipeptidyl peptidase IV produced by a *Saccharomyces cerevisiae* expression system. Biochem. Bioph Res. Co.

[bib22] Huang T., Lu Z.M., Peng M.Y., Liu Z.F., Chai L.J., Zhang X.J., Shi J.S., Li Q., Xu Z.H. (2022). Combined effects of fermentation starters and environmental factors on the microbial community assembly and flavor formation of Zhenjiang aromatic vinegar. Food Res. Int..

[bib23] Huang X., Fan Y., Lu T., Kang J., Chen J. (2020). Composition and metabolic functions of the microbiome in fermented grain during light-flavor *Baijiu* fermentation. Microorganisms.

[bib24] Huang Z., Guo W., Zhou W., Li L., Xu J., Hong J., Liu H., Zeng F., Bai W., Liu B., Ni L., Rao P., Lv X. (2019). Microbial communities and volatile metabolites in different traditional fermentation starters used for *Hong Qu* glutinous rice wine. Food Res. Int..

[bib25] Jiang L., Mu Y., Wei S., Mu Y., Zhao C. (2020). Study on the dynamic changes and formation pathways of metabolites during the fermentation of black waxy rice wine. Food Sci. Nutr..

[bib26] Jiang Y., Lv X., Zhang C., Zheng Y., Zheng B., Duan X., Tian Y. (2019). Microbial dynamics and flavor formation during the traditional brewing of *Monascus* vinegar. Food Res. Int..

[bib27] Jiao W., Xie F., Gao L., Du L., Wei Y., Zhou J., He G. (2022). Identification of core microbiota in the fermented grains of a Chinese strong-flavor liquor from Sichuan. LWT--Food Sci. Technol..

[bib28] Jing T., Chen Y.X. (2015). Organopolymerization of naturally occurring Tulipalin B: a hydroxyl-functionalized methylene butyrolactone. Org. Chem. Front..

[bib29] Kim H.J., Hwang I.K., Won M.H. (2007). Vanillin, 4-hydroxybenzyl aldehyde and 4-hydroxybenzyl alcohol prevent hippocampal CA1 cell death following global ischemia. Brain Res..

[bib30] Lan X., Wang Q., Yang J., Ma L., Yu J. (2019). Neuroprotective effect of Vanillin on hypoxic-ischemic brain damage in neonatal rats. Biomed. Pharmacother..

[bib31] Linani A., Benarous K., Bou-Salah L., Yousfi M. (2021). Hispidin, Harmaline, and Harmine as potent inhibitors of bovine xanthine oxidase: gout treatment, in vitro, ADMET prediction, and SAR studies. Bioorg. Chem..

[bib32] Liu A., Peng Y., Ao X., Pan W., Liu S. (2020). Effects of *Aspergillus niger* biofortification on the microbial community and quality of Baoning vinegar. LWT--Food Sci. Technol..

[bib33] Marín R., Mejías R., Mdvg M., Rowe F., Barroso C. (2002). Headspace solid-phase microextraction analysis of aroma compounds in vinegar: Validation study. J. Chromatogr. A.

[bib34] Mendelsohn A., Larrick J. (2014). Partial reversal of skeletal muscle aging by restoration of normal NAD levels. Rejuvenation Res..

[bib35] Nongonierma A., Fitzgerald R. (2013). Inhibition of dipeptidyl peptidase IV (DPP-IV) by proline containing peptides. J. Funct.Foods.

[bib36] Poljsak B., Milisav I. (2018). Vitamin B3 forms as precursors to NAD+: are they safe?. Trends Food Sci. Technol..

[bib37] Quast C., Pruesse E., Yilmaz P., Gerken J., Schweer T., Yarza P., Peplies J., Gloeckner F. (2013). The SILVA ribosomal RNA gene database project: improved data processing and web-based tools. Nucleic Acids Res..

[bib38] Shi H., Zhou X., Yao Y., Qu A., Ding K., Zhao G., Liu S. (2022). Insights into the microbiota and driving forces to control the quality of vinegar. LWT--Food Sci. Technol..

[bib39] Shi W., Wang J., Chen F., Zhang X. (2019). Effect of *Issatchenkia terricola* and *Pichia kudriavzevii* on wine flavor and quality through simultaneous and sequential co-fermentation with Saccharomyces cerevisiae. LWT--Food Sci. Technol..

[bib40] Song J., Zhang J., Su Y., Zhang X., Li J., Tu L., Yu J., Zheng Y., Wang M. (2020). *Monascus* vinegar-mediated alternation of gut microbiota and its correlation with lipid metabolism and inflammation in hyperlipidemic rats. J. Funct.Foods.

[bib41] Sun X., Qian Q., Xiong Y., Xie Q., Yue X., Liu J., Wei S., Yang Q. (2022). Characterization of the key aroma compounds in aged Chinese Xiaoqu *Baijiu* by means of the sensomics approach. Food Chem..

[bib42] Tesfaye W., Morales M.L., Garca-Parrilla M.C., Troncoso A.M. (2002). Wine vinegar: technology, authenticity and quality evaluation. Trends Food Sci. Technol..

[bib43] Thongekkaew J., Fujii T., Masaki K., Koyama K. (2018). Evaluation of *Candida* easanensis JK8 β-glucosidase with potentially hydrolyse non-volatile glycosides of wine aroma precursors. Nat. Prod. Res..

[bib44] Trammell S., Weidemann B., Chadda A., Yorek M., Holmes A., Coppey L., Obrosov A., Kardon R., Yorek M., Brenner C. (2016). Nicotinamide riboside opposes Type 2 diabetes and neuropathy in mice. Sci. Rep..

[bib45] Tseng Y., Yang J., Chang H., Lee Y., Mau J. (2006). Antioxidant properties of methanolic extracts from monascal adlay. Food Chem..

[bib47] Wang H., Huang Y., Huang Y. (2021). Microbiome diversity and evolution in stacking fermentation during different rounds of Jiang-flavoured *Baijiu* brewing. LWT--Food Sci. Technol..

[bib48] Wang X., Guo M., Song H., Meng Q. (2020). Characterization of key aroma compounds in traditional Chinese soy sauce through the molecular sensory science technique. LWT--Food Sci. Technol..

[bib49] Wang Z., Lu Z., Shi J., Xu Z. (2016). Exploring flavour-producing core microbiota in multispecies solid-state fermentation of traditional Chinese vinegar. Sci. Rep..

[bib50] Wang Z., Lu Z., Yu Y., Li G., Shi J., Xu Z. (2015). Batch-to-batch uniformity of bacterial community succession and flavor formation in the fermentation of Zhenjiang aromatic vinegar. Food Microbiol..

[bib51] Wei D., Fan W.L., Xu Y. (2021). Identification of water-soluble peptides in distilled spent grain and its angiotensin converting enzyme (ACE) inhibitory activity based on UPLC-Q-TOF-MS and proteomics analysis. Food Chem..

[bib52] Wu L., Lu Z., Zhang X., Wang Z., Yu Y., Shi J., Xu Z. (2017). Metagenomics reveals flavour metabolic network of cereal vinegar microbiota. Food Microbiol..

[bib53] Xiao Z., Lu J.R. (2014). Strategies for enhancing fermentative production of acetoin: a review. Biotechnol. Adv..

[bib54] Xie S., Zhao C., Fan B., Zheng Y., Xia M., Tu L., Song J., Zhao X., Wang M. (2021). Metabolic network of ammonium in cereal vinegar solid-state fermentation and its response to acid stress. Food Microbiol..

[bib55] Xue J., Liu P., Guo G., Wang W., Zhang J., Wang W., Le T., Yin J., Ni D., Jiang H. (2022). Profiling of dynamic changes in non-volatile metabolites of shaken black tea during the manufacturing process using targeted and non-targeted metabolomics analysis. LWT--Food Sci. Technol..

[bib56] Xue Y., Chen H., Yang J.R., Liu M., Bangqin H., Yang J. (2018). Distinct patterns and processes of abundant and rare eukaryotic plankton communities following a reservoir cyanobacterial bloom. ISME J..

[bib57] Yam J.C., Jiang Y., Lee J., Li S., Zhang Y., Sun W., Yuan N., Wang Y.M., Yip B., Kam K.W. (2021). The association of choroidal thickening by atropine with treatment effects for myopia: two-year clinical trial of the LAMP study. Am. J. Ophthalmol..

[bib58] Yu Y., Lu Z., Yu N., Wei X., Li G., Shi J., Xu Z. (2012). HS-SPME/GC-MS and chemometrics for volatile composition of Chinese traditional aromatic vinegar in the Zhenjiang region. J. Inst. Brew..

[bib59] Zhang Y., Zhang Y., Zhou Y., Li G., Yang W., Feng X. (2019). A review of pretreatment and analytical methods of biogenic amines in food and biological samples since 2010. J. Chromatogr. A.

[bib60] Zhang Q., Zhao C., Wang X., Li X., Zheng Y., Song J., Xia M., Zhang R., Wang M. (2020). Bioaugmentation by *Pediococcus acidilactici* AAF1-5 improves the bacterial activity and diversity of cereal vinegar under solid-state fermentation. Front. Microbiol..

[bib61] Zhao G., Kuang G., Li J., Hadiatullah, Chen Z., Wang X., Yao Y., Pan Z., Wang Y. (2020). Characterization of aldehydes and hydroxy acids as the main contribution to the traditional Chinese rose vinegar by flavor and taste analyses. Food Res. Int..

[bib62] Zhao W., Qian M., Dong H., Liu X., Bai W., Liu G., Lv X. (2022). Effect of *Hong Qu* on the flavor and quality of Hakka yellow rice wine (*Huangjiu*) produced in Southern China. LWT--Food Sci. Technol..

[bib63] Zheng Y., Mou J., Niu J., Yang S., Chen L., Xia M., Wang M. (2018). Succession sequence of lactic acid bacteria driven by environmental factors and substrates throughout the brewing process of Shanxi aged vinegar. Appl. Microbiol. Biotechnol..

[bib64] Zhu Y., Zhu, Zhang F., Zhang C., Yang L., Fan G., Xu Y., Sun B., Li X. (2018). Dynamic microbial succession of Shanxi aged vinegar and its correlation with flavor metabolites during different stages of acetic acid fermentation. Sci. Rep..

