# Supplementary material for: The dynamics of microbial community and flavor metabolites during the acetic acid fermentation of Hongqu aromatic vinegar
Source: Curr Res Food Sci. 2022 Oct 4;5:1720–31. doi: 10.1016/j.crfs.2022.10.002 (PMC9550536; doi:10.1016/j.crfs.2022.10.002)
Supplement: Multimedia component 1 [file mmc1.doc]

**Supplementary Material-1**

**Supplementary Figure. S1.** PCA in negative ion mode. Clustering (A), principal component analysis (B), and principal component scatter diagram (C).


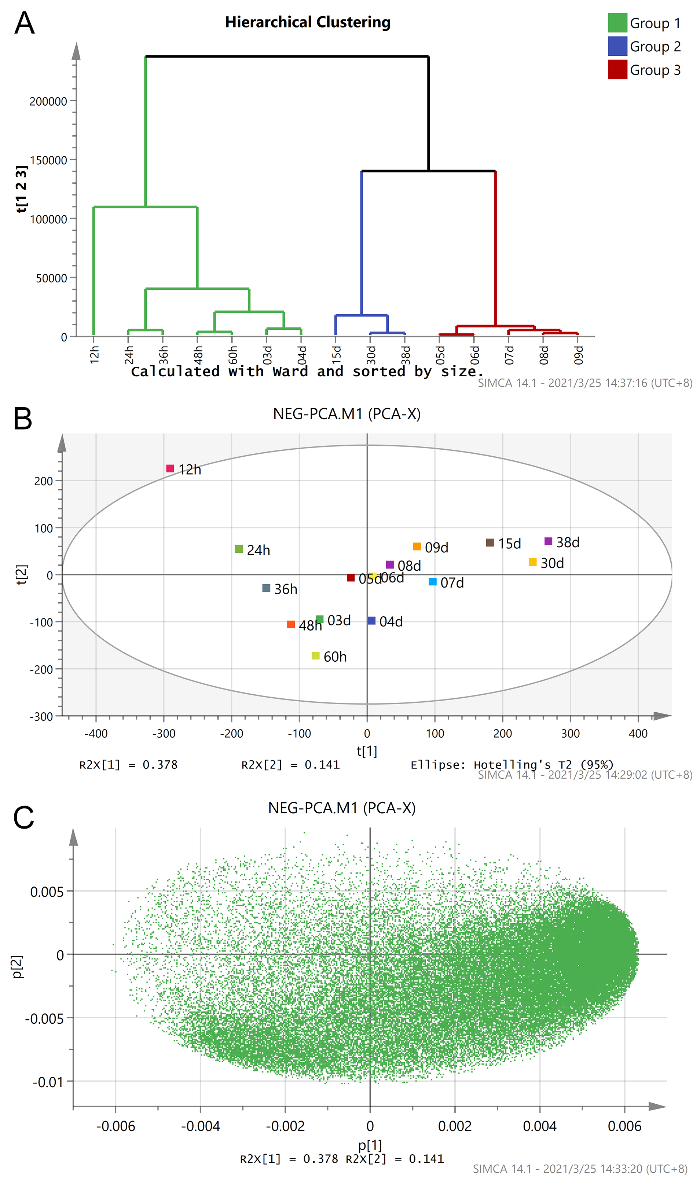


**Supplementary Figure. S2.** PCA in positive ion mode. Clustering (A), principal component analysis (B), and principal component scatter diagram (C).

**
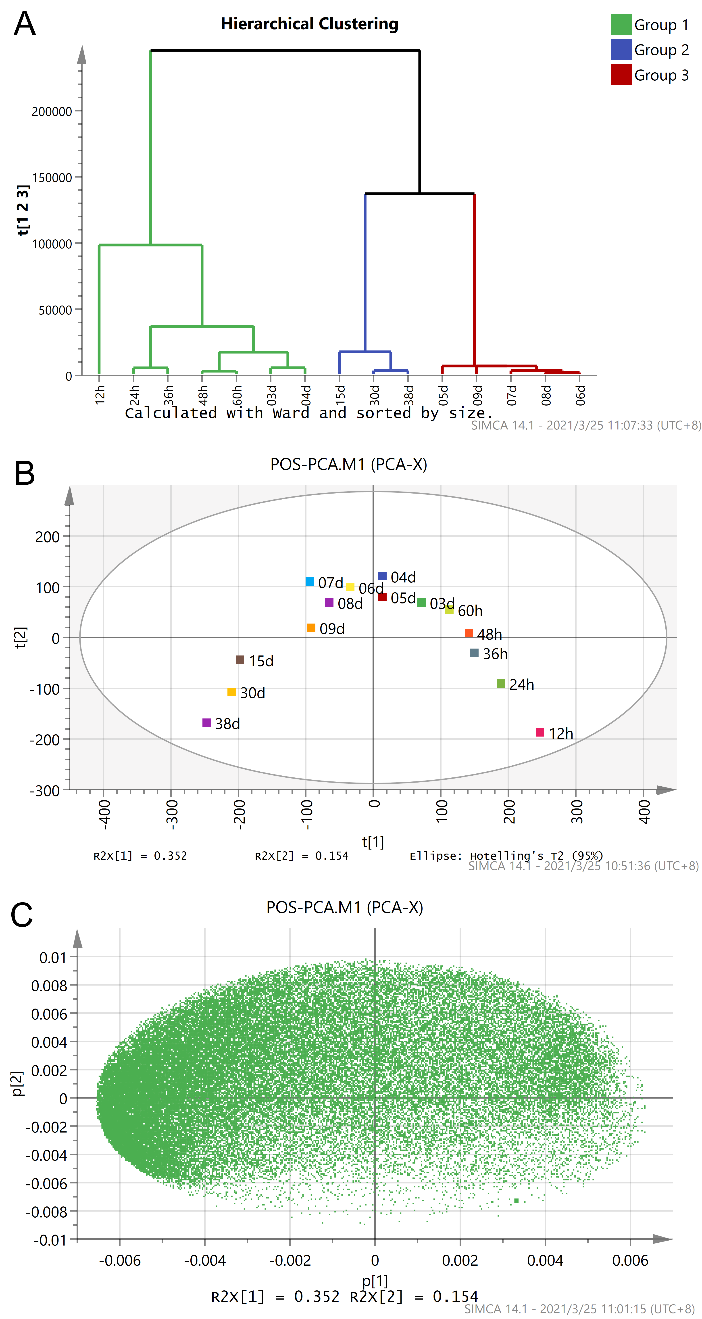
**

**Table S1** Dipeptide analysis detected in positive mode

| **No.** | **Number** | **Dipeptide** | **Sequence** | **Sensory efficacy** | **Bioactive function** | **Reference** |
| --- | --- | --- | --- | --- | --- | --- |
| 1 | POS146 | Leu-Leu | LL | bitter | DPP IV inhibitor; Glucose uptake stimulating |  |
| 2 | POS193 | Val-Glu | VE | bitter; umami; sour | ACE inhibitor; DPP IV inhibitor;  alpha-glucosidase inhibitor |  |
| 3 | POS205 | Phe-Val | FV | bitter | - |  |
| 4 | POS224 | Phe-Phe | FF | bitter | ACE inhibitor; DPP IV inhibitor |  |
| 5 | POS307 | Ala-Phe | AF | bitter | ACE inhibitor; DPP IV inhibitor |  |
| 6 | POS364 | Ile-Lys | IK | bitter |  |  |
| 7 | POS369 | Pro-Asn | PN | - | DPP IV inhibitor |  |
| 8 | POS382 | Ser-Arg | SR | - | - | - |
| 9 | POS385 | Asp-Arg | DR | - | DPP IV inhibitor; ACE inhibitor |  |
| 10 | POS409 | Val-Arg | VR | salty enhancing | ACE inhibitor; DPP IV inhibitor |  |
| 11 | POS413 | Ile-Arg | IR | - | ACE inhibitor; antioxidative; renin inhibitor;  CaMPDE inhibitor; DPP IV inhibitor |  |
| 12 | POS459 | Phe-Ser | FS | - | - | - |
| 13 | POS491 | Thr-Arg | TR | - | DPP IV inhibitor |  |
| 14 | POS511 | Val-Lys | VK | - | ACE inhibitor; DPP IV inhibitor |  |
| 15 | POS527 | Thr-Phe | TF | - | ACE inhibitor; DPP IV inhibitor;  renin inhibitor; DPP III inhibitor |  |
| 16 | POS536 | Arg-Val | RV | salty enhancing | DPP-III inhibitor |  |
| 17 | POS539 | Ala-Val | AV | - | DPP IV inhibitor; ACE inhibitor |  |
| 18 | POS549 | Ser-Leu | SL | bitter | DPP IV inhibitor |  |
| 19 | POS577 | Leu-Glu | LE | bitter | - |  |
| 20 | POS578 | Arg-Ile | RI | - | DPP IV inhibitor |  |
| 21 | POS608 | Leu-Gln | LQ | - | ACE inhibitor |  |
| **No.** | **Number** | **Dipeptide** | **Sequence** | **Sensory efficacy** | **Bioactive function** | **Reference** |
| 22 | POS614 | Ala-His | AH | umami; sour | ACE inhibitor; antioxidative; DPP IV inhibitor |  |
| 23 | POS620 | Pro-Arg | PR | bitter | ACE inhibitor; DPP III inhibitor |  |
| 24 | POS129 | Ile-Pro | IP | bitter | ACE inhibitor; DPP IV inhibitor |  |
| 25 | POS132 | His-Gly | HG | - | ACE inhibitor |  |
| 26 | POS170 | Pro-Val | PV | - | DPP IV inhibitor |  |
| 27 | POS183 | Phe-Asp | FD | - | - | - |
| 28 | POS187 | His-Thr | HT | - | DPP IV inhibitor |  |
| 29 | POS236 | Tyr-Pro | YP | bitter | ACE inhibitor; DPP IV inhibitor;  alpha-glucosidase inhibitor |  |
| 30 | POS294 | Val-Val | VV | umami; bitter | DPP IV inhibitor |  |
| 31 | POS304 | Phe-Pro | FP | bitter | ACE inhibitor; DPP IV inhibitor |  |
| 32 | POS321 | Ala-Pro | AP | - | DPP IV inhibitor; ACE inhibitor |  |
| 33 | POS325 | Pro-Ser | PS | - | DPP IV inhibitor |  |
| 34 | POS362 | Val-Trp | VW | - | ACE inhibitor; antioxidative;  DPP IV inhibitor; alpha-glucosidase inhibitor |  |
| 35 | POS388 | Pro-Glu | PE | umami | DPP III inhibitor; alpha-glucosidase inhibitor |  |
| 36 | POS408 | Pro-Thr | PT | - | ACE inhibitor; DPP IV inhibitor |  |
| 37 | POS447 | Leu-Tyr | LY | bitter | ACE inhibitor; antioxidative; renin inhibitor |  |
| 38 | POS483 | Val-Tyr | VY | bitter | ACE inhibitor; antioxidative;  DPP IV inhibitor; DPP III inhibitor |  |
| 39 | POS520 | Lys-Pro | KP | bitter | ACE inhibitor; antioxidative; DPP IV inhibitor |  |
| 40 | POS621 | Glu-His | EH | - | DPP IV inhibitor |  |
| 41 | POS125 | Gly-Leu | GL | bitter | ACE inhibitor; DPP IV inhibitor |  |

Table S1: DPP III: dipeptidyl peptidase III; DPP IV: dipeptidyl peptidase IV; CaMPDE: calmodulin-dependent phosphodiesterase.

**Reference**
